# MEF2C-MYOCD and Leiomodin1 Suppression by miRNA-214 Promotes Smooth Muscle Cell Phenotype Switching in Pulmonary Arterial Hypertension

**DOI:** 10.1371/journal.pone.0153780

**Published:** 2016-05-04

**Authors:** Sanghamitra Sahoo, Daniel N. Meijles, Imad Al Ghouleh, Manuj Tandon, Eugenia Cifuentes-Pagano, John Sembrat, Mauricio Rojas, Elena Goncharova, Patrick J. Pagano

**Affiliations:** 1 Department of Pharmacology and Chemical Biology University of Pittsburgh, Pittsburgh, Pennsylvania, 15261, United States of America; 2 Vascular Medicine Institute, University of Pittsburgh, Pittsburgh, Pennsylvania, 15261, United States of America; 3 Division of Pulmonary, Allergy and Critical Care Medicine, University of Pittsburgh, Pittsburgh, Pennsylvania, 15261, United States of America; William Harvey Research Institute, Barts and The London School of Medicine and Dentistry, Queen Mary University of London, UNITED KINGDOM

## Abstract

**Background:**

Vascular hyperproliferative disorders are characterized by excessive smooth muscle cell (SMC) proliferation leading to vessel remodeling and occlusion. In pulmonary arterial hypertension (PAH), SMC phenotype switching from a terminally differentiated contractile to synthetic state is gaining traction as our understanding of the disease progression improves. While maintenance of SMC contractile phenotype is reportedly orchestrated by a MEF2C-myocardin (MYOCD) interplay, little is known regarding molecular control at this nexus. Moreover, the burgeoning interest in microRNAs (miRs) provides the basis for exploring their modulation of MEF2C-MYOCD signaling, and in turn, a pro-proliferative, synthetic SMC phenotype. We hypothesized that suppression of SMC contractile phenotype in pulmonary hypertension is mediated by miR-214 *via* repression of the MEF2C-MYOCD-leiomodin1 (LMOD1) signaling axis.

**Methods and Results:**

In SMCs isolated from a PAH patient cohort and commercially obtained hPASMCs exposed to hypoxia, miR-214 expression was monitored by qRT-PCR. miR-214 was upregulated in PAH- vs. control subject hPASMCs as well as in commercially obtained hPASMCs exposed to hypoxia. These increases in miR-214 were paralleled by MEF2C, MYOCD and SMC contractile protein downregulation. Of these, LMOD1 and MEF2C were directly targeted by the miR. Mir-214 overexpression mimicked the PAH profile, downregulating MEF2C and LMOD1. AntagomiR-214 abrogated hypoxia-induced suppression of the contractile phenotype and its attendant proliferation. Anti-miR-214 also restored PAH-PASMCs to a contractile phenotype seen during vascular homeostasis.

**Conclusions:**

Our findings illustrate a key role for miR-214 in modulation of MEF2C-MYOCD-LMOD1 signaling and suggest that an antagonist of miR-214 could mitigate SMC phenotype changes and proliferation in vascular hyperproliferative disorders including PAH.

## Introduction

Pulmonary Arterial Hypertension (PAH) is a complex and progressive disease characterized by increased lung vascular resistance and pressure, which leads to right ventricular failure and death. A major hallmark of PAH is irreversible pulmonary arterial remodeling, manifested by excessive cellular proliferation, apoptotic resistance and abnormal contractile-to-synthetic vascular smooth muscle cell (VSMC) phenotype switching[1–3]. Sustained VSMC de-differentiation underlies a characteristic vascular proliferative phase in PAH, which propagates vessel wall occlusion. Although diverse environmental signals including sustained hypoxia and alterations in extracellular matrix are known to regulate SMC phenotype [4–6], the mechanisms that control SMC de-differentiation are not well understood.

Myocardin (MYOCD), a SMC-restricted transcriptional coactivator of serum response factor (SRF), physically interacts with SRF and selectively induces expression of all hallmark CArG-dependent SMC marker genes, including smooth muscle-actin (SMA), SM-myosin heavy chain (MYH11), SM22, and calponin1 (CNN1) [7–12]. The molecular basis of MYOCD-induced transcriptional activation is complex involving additional regulation via myocyte enhancer factor 2C (MEF2C)[13] which serves as upstream coordinator of SMC differentiation, *i*.*e*. the contractile phenotype[14].

The VSMC contractile state is characterized by expression of SMC-specific genes, *i*.*e*. MYH11, smoothelin, CNN1 among others controlled by MYOCD [7–12,15]. Leiomodin1 (LMOD1) is a recently described SMC-restricted gene transcriptionally regulated by MYOCD and, similar to other MYOCD-regulated genes, LMOD1 is expected to play a fundamental role in SMC contractility [16]. Despite the importance of MEF2C-MYOCD modulation in SMC phenotype switching, incomplete knowledge of MEF2C, MYOCD and SMC-specific proteins in PAH limits our understanding of the underlying mechanisms controlling differentiation.

Epigenetic regulation by microRNAs (miRs) has garnered significant intrigue in recent years. Short non-coding RNAs that regulate gene expression through translational repression or degradation of mRNA[17], miRs increasingly are shown to play a role in cardiovascular physiology and pathophysiology[18], including PAH[19,20]. A number of miRs, such as miR-21[21] and miR-143/145[19,22], have been shown to modulate smooth muscle cell phenotype and proliferation. The miR and mechanisms, however, involved in the control of MEF2C-MYOCD and LMOD1-mediated SMC remodeling are unknown. miR-214, a member of the miR-199a-214 cluster, is implicated in cancer progression[23], skeletal muscle differentiation[24,25], ischemia injury[26], mitochondrial fatty acid oxidation[27], and failing human hearts[28]. In the vasculature, expression of miR-214 is ubiquitous [29], but its role in phenotype control is entirely unexplored.

In the current study, we explored the potential for miR-214 to alter SMC phenotype at multiple levels, i.e. MEF2C, MYOCD and LMOD1 expression. Herein, we demonstrate that lung explant-derived human PAH-PASMC and control hPASMC exposed to chronic hypoxia exhibited markedly upregulated miR-214. This coincided with attenuated MEF2C expression, marked decreases in MYOCD, LMOD1, MYH11, smoothelin and CNN1, and increased cell proliferation, all of which were abrogated by anti-miR-214. Thus, we propose a novel mechanism directly linking miR-214 in a pleiotropic suppression of SMC differentiation.

## Method

A full methods section is available in the supplement file (See S1 File for more details).

### Human Samples

The study was approved by the University of Pittsburgh Institutional Review Board and written informed consent was obtained from all participating individuals. Lung and Pulmonary Artery (proximal/second branch) tissues from non-diseased subjects and idiopathic PAH patients undergoing lung transplant were provided by the PACCM Bio-Bank under protocols approved by the University of Pittsburgh Institutional Review Board. Pulmonary artery smooth muscle cells (hPASMCs) from non-diseased rejected transplants and PAH explants (henceforth designated as “non-PAH controls” and “PASMCs derived from PAH patients”) were isolated and characterized as described previously[30]. Each experiment was repeated using primary (passages 3–8) hPASMCs from at least three non-PAH and PAH subjects.

### Cell Culture

Human pulmonary artery smooth muscle cells (hPASMC) were purchased from Lonza (Walkersville, MD) and are hereafter designated as “control PASMCs”. All hPASMCs were cultured in growth media SmGM-2 (Lonza) in 5% fetal bovine serum (FBS). The media was reduced to 0.2% FBS for serum deprivation. Cells were grown to ∼80–90% confluence, and then subjected to normoxia (21% O_2_, 5% CO_2_, balance N_2_) or hypoxia (1% O_2_, 5% CO_2_, balance N_2_) as we described previously [31]. HEK293 cells were grown in DMEM supplemented with 10% FBS (Gibco, Carlsbad, CA).

### Oligonucleotide Transfection

Transfections with miR-214 mimic, anti-miR-214 (miR-214 antagomiR) or scrambled control (Invitrogen Inc.) at a final concentration of 50nmol/L were performed using Lipofectamine 2000 reagent following the manufacturer’s recommendations.

### Quantitative Reverse Transcriptase—PCR

Total RNA was extracted from human lung tissues and hPASMC by using miRNeasy Mini kit (Qiagen). For RT-PCR, total RNA was reverse transcribed to cDNA by using Superscript III (Invitrogen *Inc*.) following the manufacturer’s protocol. For microRNA and gene expression analysis, we used Taqman probe and primer sets available from Invitrogen *Inc*. RNU43 were used as internal control for the expression of miR-214 in human samples. mRNA expression of all genes included in this study are relative to 18s and the results are presented as fold changes in gene expression calculated using the 2^-ΔΔ ct^ method.

### Luciferase Reporter Assay

The constructs containing 3’ untranslated region (3’-UTR) of LMOD1 and MEF2C, including the putative miR-214 binding sequence cloned into a luciferase reporter was obtained from Switchgear Genomics^™^, and GeneCoepia^™^, respectively. To test the binding specificity, miR-214 binding sites in the luciferase constructs of both LMOD1 (2 sites) and MEF2C (1 site) were mutated using the Site-Directed Mutagenesis Kit (Agilent Technologies). For the two different miR-214 binding sites on LMOD1 3’UTR, we generated plasmid constructs containing both a single-site mutant as well as one carrying mutations on both the binding sites (double mutant). For luciferase reporter assay, cells (HEK293 or hPASMCs) were seeded in 24-well plates and co-transfected with 3’-UTR reporter plasmid (wildtype or mutants), and miR-214 mimic or non-targeting mimic control. hPASMCs were transfected using Cytofect ^™^ Smooth Muscle Transfection kit, (Cell Applications, Inc.). Forty-eight hours after transfection, Luciferase Assay Reagent was added to measure luciferase activity.

### Statistical Analyses

Data are expressed as mean ± SEM, and for comparison of results between two data sets, an unpaired Students’ *t* test was performed. One-way ANOVA followed by Bonferroni multiple comparison tests were used for comparison of results among more than two groups using Graphpad Prism software (version 5.04). p<0.05 was considered statistically significant.

## Results

### LMOD1, MYOCD & MEF2C Expression are Downregulated in PAH

**Fig 1:**
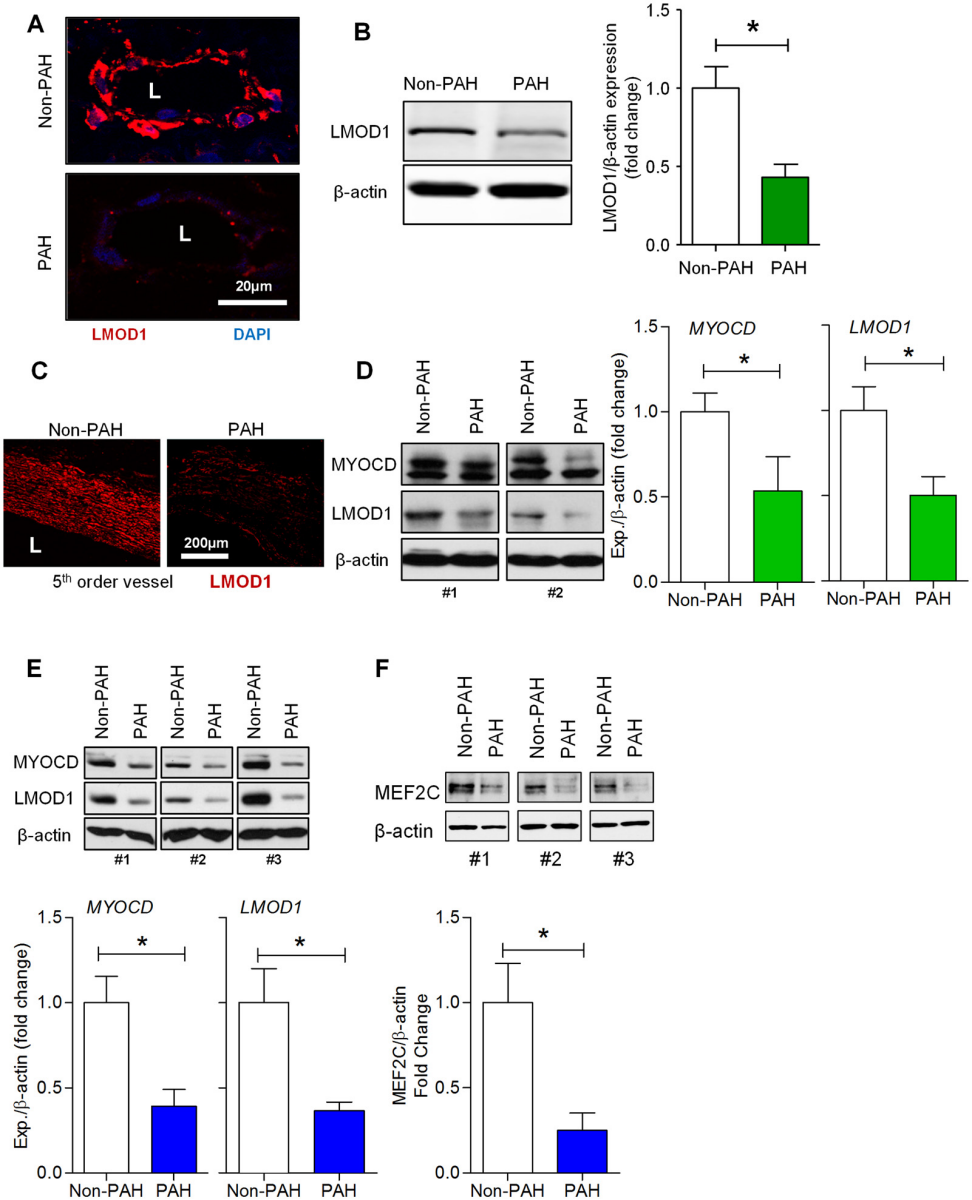
Contractile proteins, leiomodin1 (LMOD1), myocardin (MYOCD) and MEF2C are downregulated in pulmonary artery smooth muscle cells (hPASMCs) in pulmonary arterial hypertension (PAH). (A,B) LMOD1 expression in lungs from PAH and non-PAH patients (n = 3) was determined by immunofluorescence and Western blot. Images from lung sections showed a marked decrease in LMOD1 expression (red fluorescence) in the vessel wall, primarily the media, of PAH samples compared to non-PAH group; DAPI nuclear staining (blue), (**A**). Western blot analysis of total lung homogenates showed a significant decrease in LMOD1 expression in PAH compared to control lung homogenates (**B**). (C-D) LMOD1 expression in the pulmonary artery (PA) from PAH and non-PAH patients (*n = 3*) was determined using immunofluorescence and Western blot analysis, respectively. Immunofluorescence images from PA sections of non-PAH samples showed expression of LMOD1 specific to the medial layer (smooth muscle layer, red fluorescence). LMOD1 expression was significantly attenuated in PAH samples compared to non-PAH group (**C**). Western blot analysis of total PA homogenates showed significant decreases in myocardin (MYOCD) and LMOD1 expression in PAH compared to non-PAH tissue homogenates (**D**). Western blot analysis of PASMCs-derived from PAH and non-PAH subjects showed decreased levels of MYOCD and LMOD1 protein in PAH group compared to non-PAH group (*n = 3–5*) (**E**). Western blot analysis of PASMCs-derived from PAH and non-PAH patients showed decreased levels of MEF2C protein in the PAH group compared to non-PAH subjects (*n = 4*) (**F**). Blots are representative; graphs depict mean ± SEM (*, p<0.05).

Similar to contractile proteins under the direct transcriptional activation of MYOCD and their diminished presence in vascular remodeling, we hypothesized that LMOD1 expression would be attenuated in human PAH. To test this, we first sought to determine the levels of expression of LMOD1 in human lungs, PA and hPASMCs derived from PAH patients. Immunofluorescence (IF) revealed that LMOD1 was exclusively expressed in the media of PAs from control human lungs, and its expression was decreased in the PAH cohort (Fig 1A). Western blot of lung homogenates showed expression was attenuated ~ 50% in PAH (Fig 1B). IF and Western blot of PA isolated from human lungs (Fig 1C and 1D) further supported our observation that LMOD1 is expressed exclusively in the media and significantly attenuated in PAH patients vs. controls. To interrogate these findings at the cellular level, we examined LMOD1 in primary hPASMCs isolated from non-PAH controls vs. PAH patients. LMOD1 was significantly suppressed in hPASMC from PAH patients (Fig 1E). These results demonstrate that expression of LMOD1, a SMC-specific contractile protein, is attenuated in human PAH.

An established regulator of LMOD1, we concomitantly investigated the expression of MYOCD. Western blot showed that MYOCD was attenuated in isolated PAs as well as in hPASMCs from PAH vs. non-PAH subjects (Fig 1D and 1E, respectively). To determine whether MEF2C, a modulator of MYOCD, is aberrantly expressed during PAH, we investigated the expression of MEF2C in hPASMCs from non-PAH and PAH subjects. Western blot analysis showed that MEF2C protein was markedly attenuated (>60%) in hPASMCs from PAH patients vs. controls (Fig 1F). qRT-PCR data showed that mRNA expression of MEF2C, MYOCD, LMOD1, MYH11, smoothelin and CNN1 was significantly attenuated in PAs of PAH patients (S1A–S1F Fig). Furthermore, IF analysis on vessel wall of lung sections of PAH and non-PAH patients showed thickening of the medial layer with PAH, suggesting extensive vascular remodeling (S2A Fig). There is a decreased expression of LMOD1 and smooth muscle α-actin, and both proteins co-localized in the medial layer (S2A Fig). Similar to LMOD1 and smooth muscle α-actin, smoothelin expression was also downregulated in the media of PAs of PAH compared to non-PAH subject lung sections (S2B Fig).

### miR-214, a Potential Regulator of LMOD1 and MEF2C, is Upregulated in Human PAH and Experimental PAH

**Fig 2:**
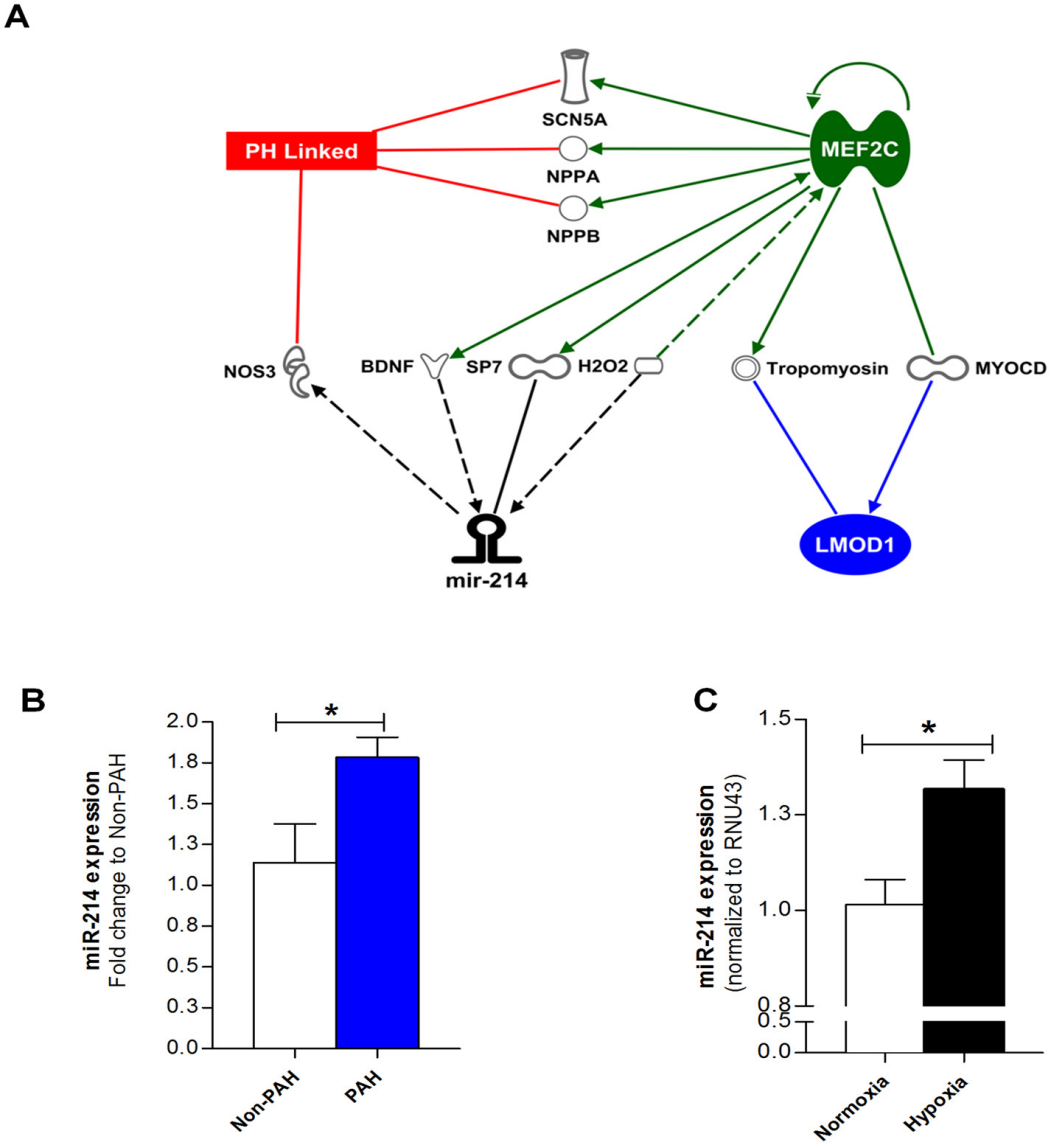
miR-214 is significantly upregulated in PAH. (**A**) Integrated Pathway Analysis (IPA) shows potential association between miR-214-MEF2C-LMOD1 in PAH, and other genes unique to PAH potentially associated with miR-214. Relationships between molecules are represented as follows: bold line, direct interaction; dotted line, indirect interaction; line with arrowhead, represents directionality of interaction; SCN5A, sodium channel, voltage gated, type V alpha subunit; NPPA, natriuretic peptide A; NPPB, natriuretic peptide B; NOS3, nitric oxide synthase, endothelial; BDNF, brain-derived neutrotrophic factor; SP7, Sp7 transcription factor; H_2_O_2_, hydrogen peroxide; MEF2C, myocyte enhancer factor 2C; MYOCD, myocardin; LMOD1, leiomodin1. (**B**) miR-214 expression is increased (~1.6-fold) in SMC-derived from PAH patients compared to controls subjects as measured by q-RT PCR (*n = 7–8*). (**C**) Serum-deprived control PASMCs were exposed to normoxia (21% O_2_) or hypoxia (1% O_2_) for 24 hrs and, miR-214 expression determined by q-RT PCR. Hypoxia resulted in significant increase in miR-214 expression in PASMCs compared to normoxic controls (*n = 8*). Graphs represent mean ± SEM. *, p<0.05 versus respective control.

We next sought to identify novel miRs that could be involved in the regulation of smooth muscle de-differentiation. Using Ingenuity Pathway Analysis (IPA), we discovered a strong association among pulmonary hypertension, MEF2C, LMOD1, and miR-214 (Fig 2A). To interrogate the validity of miR-214 as a phenotypic modulator, we first employed qRT-PCR to determine the expression of miR-214 in hPASMCs from non-PAH and PAH subjects. As shown in Fig 2B, miR-214 expression was increased ~1.6-fold in SMCs isolated from PAH patients compared to non-PAH controls. We investigated miR-214 expression in cultured hPASMCs (Lonza) exposed to hypoxia (known to mimic clinical features of PAH). miR-214 was significantly upregulated in hPASMCs exposed hypoxia vs. normoxia (Fig 2C).

### MEF2C and LMOD1 are Novel Direct Targets of miR-214

**Fig 3:**
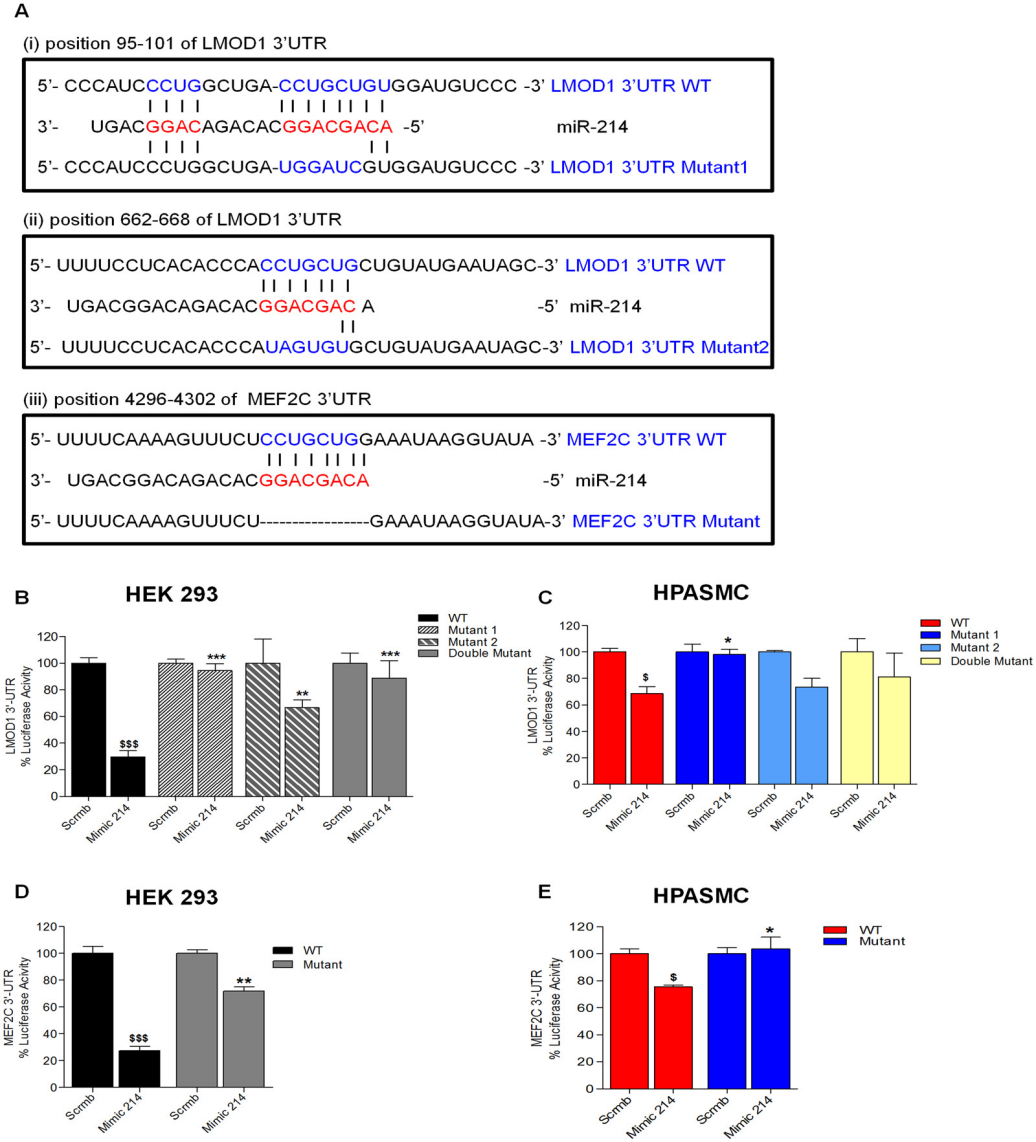
LMOD1 and MEF2C are validated as a direct target of miR-214. (**A**) miR-214 binding sites in the 3’-UTR of LMOD1 and MEF2C transcripts. Seed and target sequences are in blue, and base-pairing between miR-214 and target site marked by vertical lines. Mutations in the reporter plasmid construct are indicated in red letters. The double mutant (not shown) carries mutations to both sites in the 3’UTR construct vs. the single site mutants shown in 3A. (**B-E**) For luciferase assays, HEK293 cells (B, D) or hPASMCs (C, E) were transfected with miR-214 mimic or non-targeting mimic control and wildtype vs. mutant constructs for LMOD1 (B, C) or MEF2C (D, E). Relative luciferase activities were determined 48 hrs post transfection. (*n = 3–5*), ^$^, p<0.05 WT scrmb vs. mimic; *, p<0.05 WT mimic vs. mutants mimic.

We applied TargetScan^™^ prediction algorithms to explore whether miR-214 could act as a regulator of LMOD1 and MEF2C. As shown in Fig 3A, we identified miR-214 putative binding sites (bp 95–101; bp 662–668) in the 3’UTR of LMOD1 gene, and one in MEF2C (bp 4296–4302). Both LMOD1 and MEF2C 3’UTRs display complete 7-bp complementarity to the seed region of miR-214, and these are conserved among human, mouse, rat and other vertebrates (Targetscan ver 6.0; S3 Fig). To determine whether the negative regulatory effects of miR-214 on LMOD1 and MEF2C expression were mediated through binding of miR-214 to the predicted sites in their respective 3’ UTRs, we performed miR-target reporter assay using a chimeric construct containing the 3’UTR downstream of the luciferase ORF in HEK293 cells. Indeed, for both LMOD1 (Fig 3B) and MEF2C (Fig 3D), a marked reduction (~75%) in luciferase activity in cells transfected with miR-214 mimic compared to non-targeting control was observed. Further, we constructed mutants of the 3’UTR reporter by mutating either or both miR-214 binding sites on the LMOD1 3’UTR (single-site or double mutants) as well as the singular site on MEF2C 3’ UTR. Luciferase reporter assay performed in both HEK293 (Fig 3B and 3D) and hPASMCs (Fig 3C and 3E) demonstrated that loss of binding sites on the 3’UTR abrogated the effect of miR-214 mimic. Interestingly, it also shows that the miR-214 binding site further from the 3’ region of LMOD1 gene (site 2) is only partially capable of rescuing the effect of miR-214, suggesting a stronger effect of miR-214-mediated suppression *via* site 1 on LMOD1 3’UTR. These results establish that miR-214 interacts with the LMOD1 and MEF2C 3’UTR exerting post-transcriptional gene regulation.

### miR-214 Negatively Regulates the Expression of MEF2C and Smooth Muscle Cell Contractile Genes

**Fig 4:**
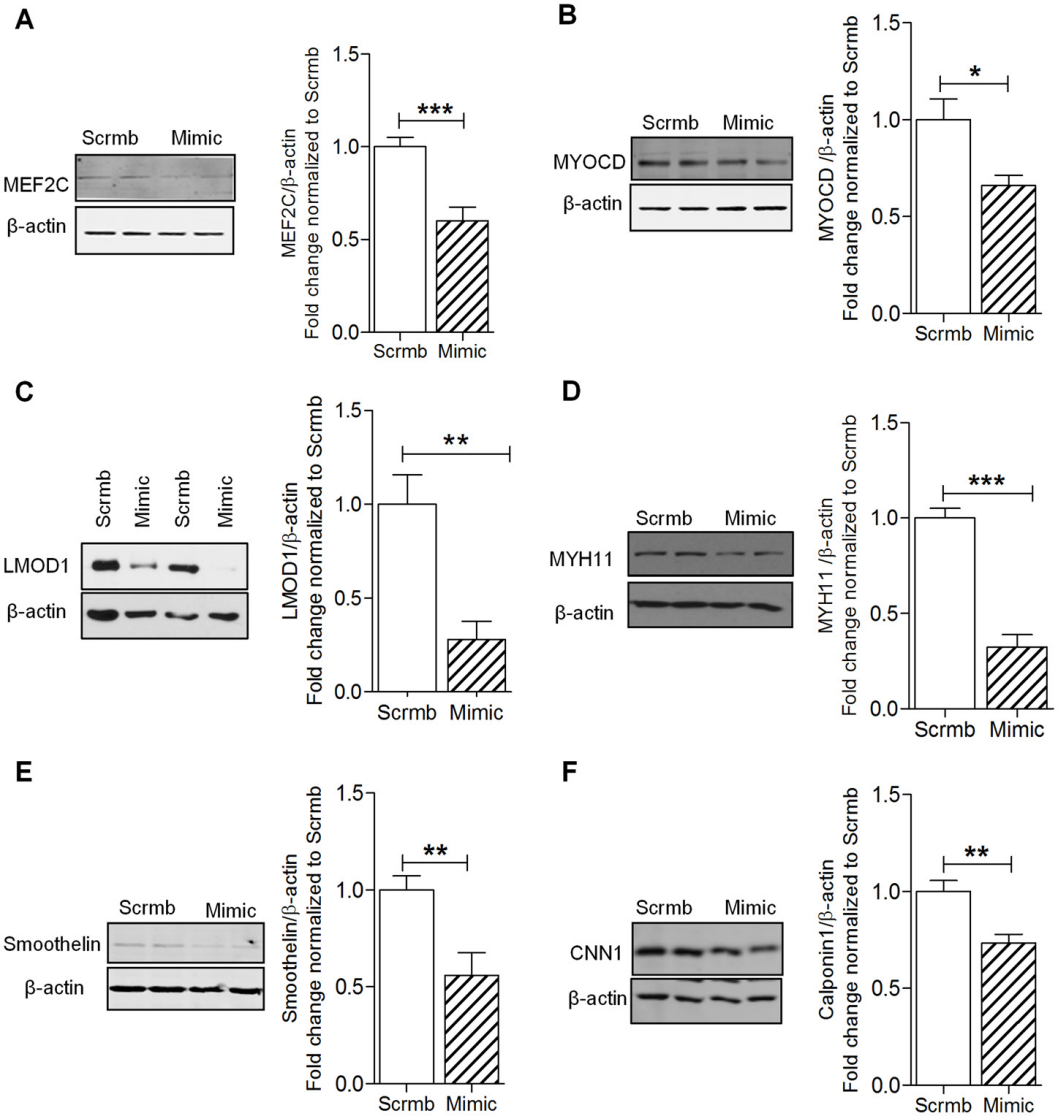
miR-214 regulates expression of SMC contractile genes. HPASMCs were transfected with miR-214 (Mimic) or scrambled control (Scrmb) under normoxic conditions for 72 hrs and lysed for Western blot analysis. Overexpression of miR-214 using its mimic significantly decreased the expression of MEF2C (**A**), MYOCD (**B**), LMOD1 (**C**), MYH11 (**D**), smoothelin (**E**), CNN1 (**F**), (*n = 4–9*). Graphs represent mean ± SEM (*, p<0.05, **, p<0.01, ***, p<0.001).

The abovementioned results illustrate that miR-214 is upregulated in human PAH and hypoxia, and previous reports demonstrate that contractile genes are downregulated in response to environmental factors and diseases [1,32], leading to profound cellular changes. To recapitulate the hypoxia-driven SMC phenotypic changes and validate the predicted role of miR-214, we performed gain- and loss-of-function experiments interrogating the expression of SMC contractile proteins, which might be modulated in PAH. Under normoxic conditions, overexpression of miR-214 in hPASMCs with miR-214 mimic reduced MEF2C and MYOCD expression by ~30% compared to scrambled control (Fig 4A and 4B). Furthermore, nearly 50–70% reduction of LMOD1, MYH11 and smoothelin protein was observed in hPASMCs transfected with mimic (Fig 4C–4E). Expression of CNN1 protein was reduced by ~30% compared to scrambled control upon overexpression of miR-214 mimic (Fig 4F).

**Fig 5:**
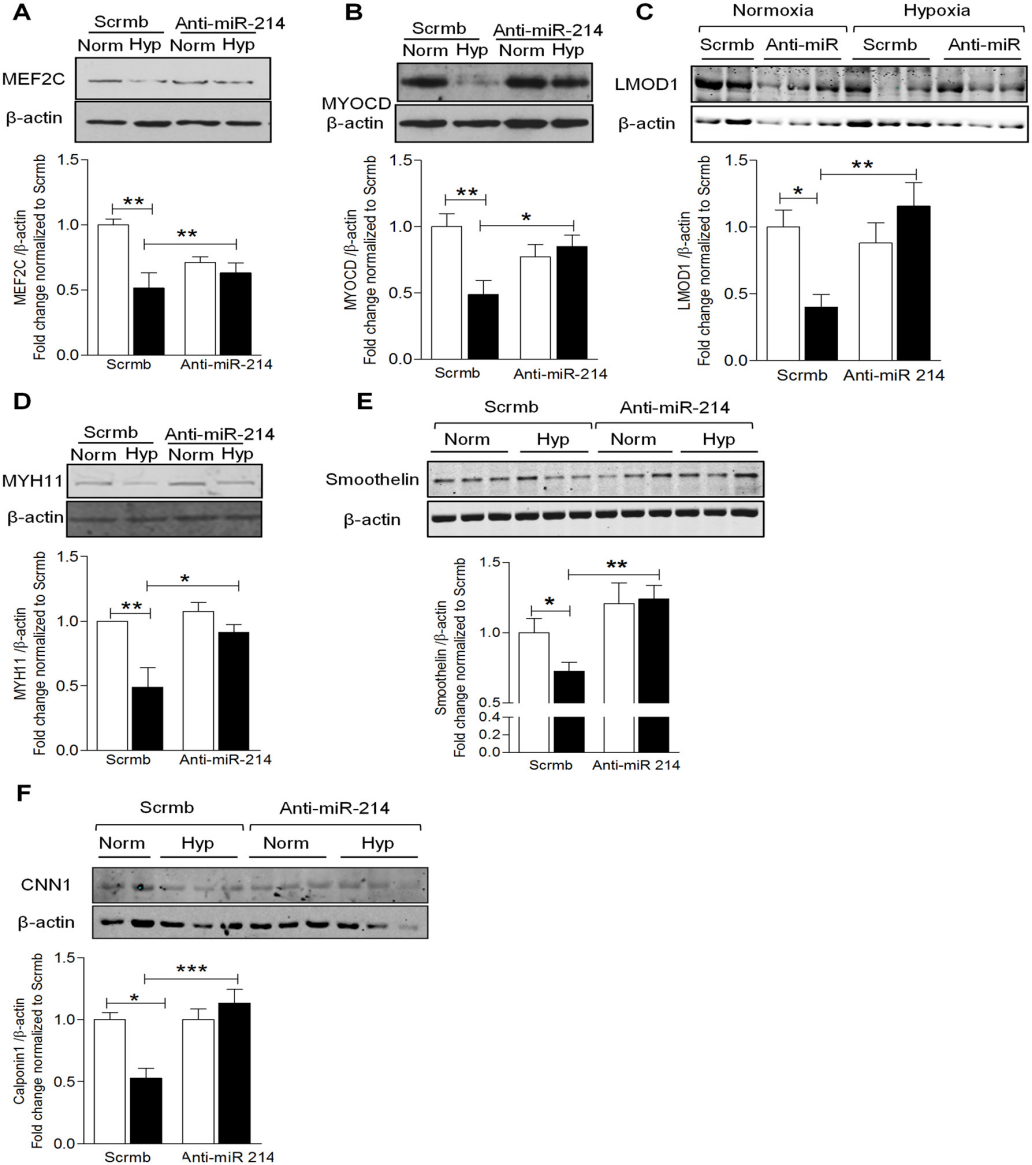
Inhibition of miR-214 restores expression of SMC contractile proteins. HPASMCs transfected with anti-miR-214 or scrambled control (Scrmb) were exposed to normoxia (open bars) or hypoxia (filled bars) for 24 hrs and lysed for Western blot. Hypoxia significantly decreased the expression of contractile proteins, while transfection with anti-miR-214 significantly reversed the hypoxia-induced suppression of contractile proteins, MEF2C (**A**), MYOCD (**B**), LMOD1 (**C**), MYH11(**D**), smoothelin (**E**) and CNN1 (**F**), (*n = 3–6*). Graphs represent mean ± SEM (*, p<0.05, **, p<0.01, ***, p<0.001).

To examine whether miR-214 antagonism is able to reverse the hypoxia-induced phenotypic switching, we transfected hPASMCs with antagomiR (anti-miR-214). As illustrated in Fig 5A and 5B, exposing hPASMCs to hypoxia significantly reduced expression of MEF2C and MYOCD by ~50% while transfection with anti-miR-214 reciprocally reversed the hypoxia-induced downregulation. Further, hypoxia significantly reduced expression of SMC-specific contractile gene, LMOD1, by ~50% and loss-of-function with anti-miR-214 completely reversed the hypoxia-induced LMOD1 downregulation (Fig 5C). As MYH11, smoothelin and CNN1 are under the control of MEF2C and MYOCD, we interrogated changes in their expression in response to miR-214. Hypoxia reduced MYH11 (~50%), smoothelin (~30%) and CNN1 (~50%) expression, and anti-miR-214 reversed this suppression (Fig 5D–5F). In the case of smoothelin, reversal exceeded baseline control levels, suggesting that miR-214 might play a role in regulating basal expression. In aggregate, these results suggest that miR-214 directly modulates contractile protein expression at the level of LMOD1, in addition to upstream disruption of MEF2C-MYOCD signaling.

### miR-214 Promotes Hypoxia-Induced Smooth Muscle Cell Proliferation

**Fig 6:**
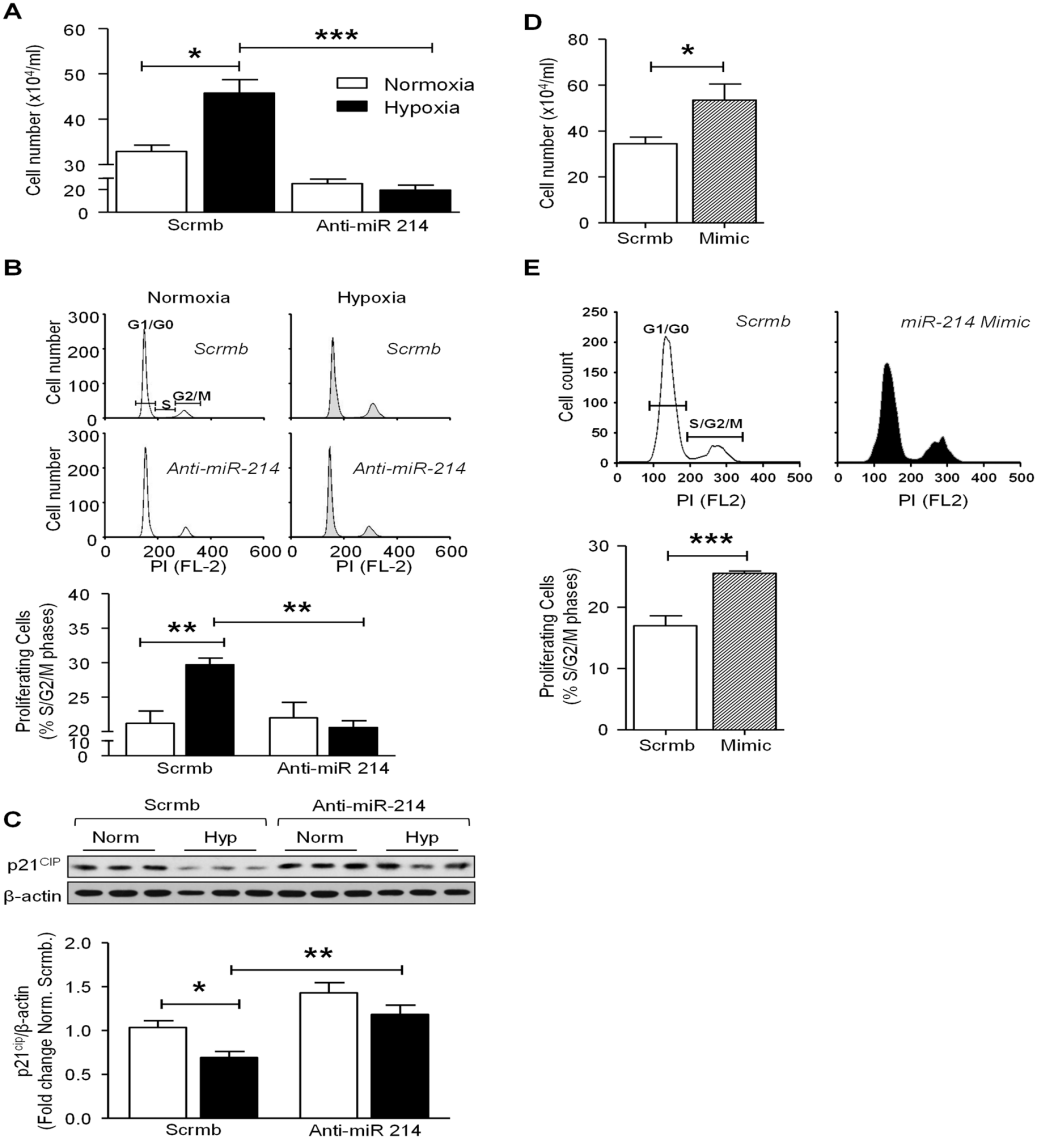
miR-214 regulates hypoxia-induced proliferation and cell cycle progression of hPASMCs. HPASMCs transfected with anti-miR-214 or scrambled control (Scrmb) were exposed to normoxia (open bars) or hypoxia (filled bars) for 24 hrs and harvested for cell counting by trypan blue exclusion (**A**), trypsinized for flow cytometry analysis using propidium iodide staining (**B**), or lysed for Western blot analysis (**C**). In both cell count and cell cycle analysis, hypoxia significantly increased the number of proliferating cells and hPASMC transfected with anti-miR-214 displayed significantly attenuated hypoxia-induced proliferation (*n = 5–6*). Also, hPASMC transfected with anti-miR-214 attenuated hypoxia-induced increase in S+ G2/M phase cells as depicted by cell cycle analysis. (**C**) Hypoxia significantly suppressed p21^*cip*^ levels in hPASMC and anti-miR-214 significantly reversed hypoxia-induced suppression of p21^*cip*^. HPASMCs were transfected with miR-214 mimic or scrambled control under normoxic conditions for 72 hrs and harvested for cell counting by trypan blue exclusion (**D**), trypsinized for flow cytometry analysis using propidium iodide staining (**E**). miR-214 mimic significantly increased the number of proliferating cells and increased the number of cells in S+ G2/M phase (D, E, respectively; *n = 5–6*). Graphs represent mean ± SEM (*, p<0.05, **, p<0.01, ***, p<0.001).

Dysregulation of SMC proliferation is central to the pathogenesis of PAH, and it is widely accepted that a terminally differentiated SMC exhibits decreased proliferative potential[1]. To determine whether miR-214 participates in the induction of hPASMC proliferation, we explored the potential for a reduced proliferative capacity of SMC transfected with anti-miR-214 under hypoxic conditions. Consistent with previous reports, we found that hypoxia significantly induced hPASMC proliferation (~1.5-fold vs. normoxia) as measured by Trypan blue exclusion (Fig 6A), cell cycle progression (Fig 6B), and BrdU incorporation (S4 Fig) methodologies. Further, miR-214 antagomiR significantly attenuated hypoxia-induced hPASMC proliferation (Fig 6A and 6B & S4 Fig). Because proliferation of cells is dependent on cell cycle re-entry, we sought to determine whether progression of cells from G1/G0 to S-phase is modulated by miR-214 in response to hypoxia. Western blot analysis revealed that hypoxia significantly attenuated the expression of p21^*cip*^ (~50% less), an effect which was abrogated in hPASMC treated with anti-miR-214 (Fig 6C). Taken together, these data suggested that under hypoxic conditions, miR-214 promotes transition into S and G2/M for which p21^*cip*^ inhibition is permissive [33]. To test this further, we investigated whether over expression of miR-214 drives proliferation by examining SMC number and cell cycle progression. When compared with control, hPASMCs treated with miR-214 mimic showed a higher cell number (~1.5-fold more) (Fig 6D) and greater cell cycle progression into S and G2/M phases (Fig 6E). This response is replicated in cells from PAH vs. non-PAH subjects, (2-fold rise; S5 Fig).

### Inhibition of miR-214 Rescues Smooth Muscle Cell Contractile Phenotype in PAH-PASMC

**Fig 7:**
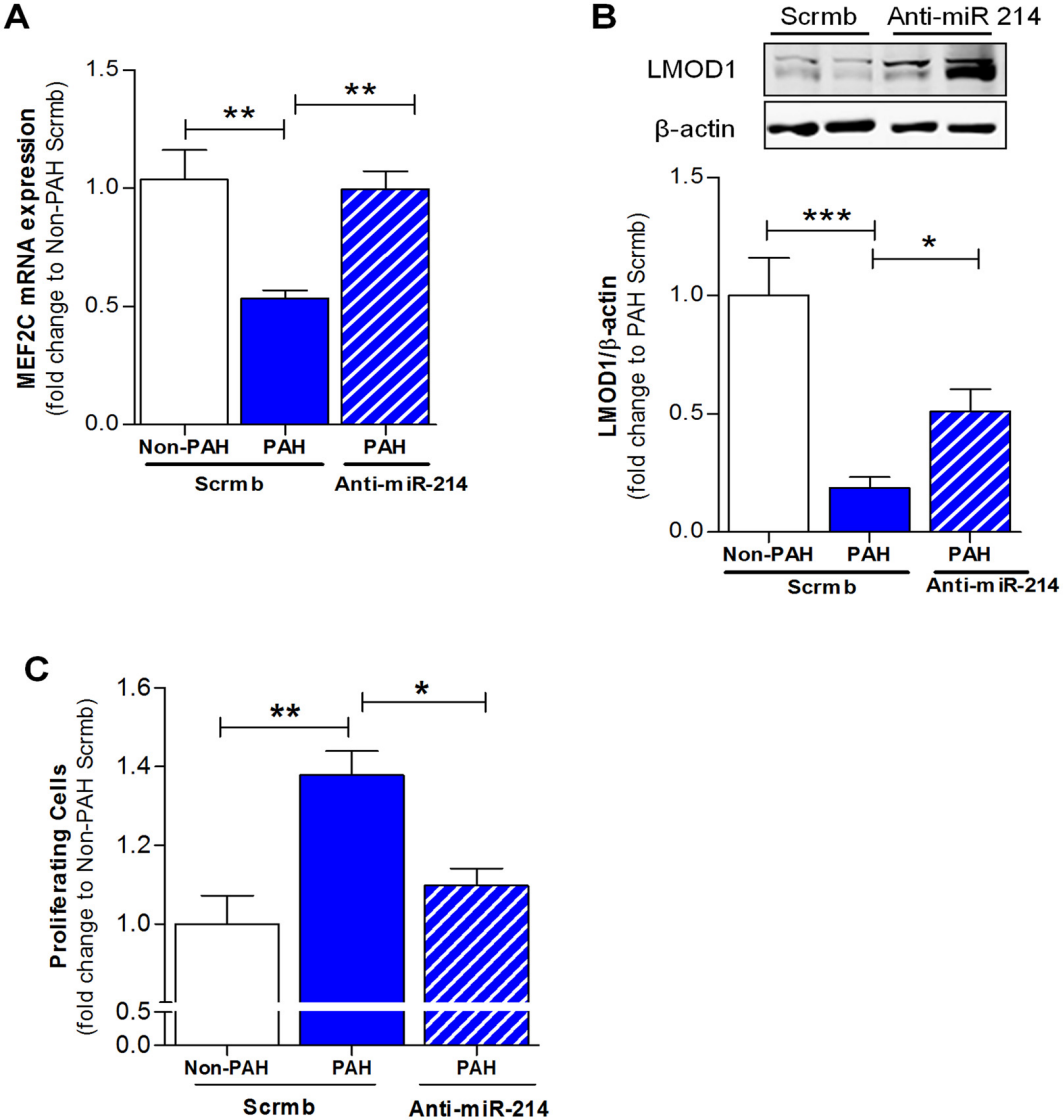
miR-214 promotes suppression of MEF2C-LMOD1 signaling axis in PAH-PASMCs. Analysis of SMC phenotypic markers in PASMCs derived from PAH patients (*n = 3–5*). PAH-PASMCs were transfected with anti-miR-214 or scrambled control (Scrmb) and lysed for q-RT PCR and Western blot, 72 hrs after transfection. miR-214 antagonism in PAH-PASMCs reversed the expression of MEF2C (**A**) and LMOD1 (**B**) comparable to non-PAH controls. (**C**) Analysis of proliferation in PASMCs derived from PAH patients (*n = 3*). PAH-PASMCs were transfected with anti-miR-214 or Scrmb and trypsinized for cell cycle analysis 72 hrs after transfection. miR-214 antagonism in PAH-PASMCs significantly inhibited PASMC proliferation. Graphs represent mean ± SEM (*, p<0.05, **, p<0.01, ***, p<0.001).

Given the aforementioned results demonstrating the effects of inhibition of miR-214 *in vitro*, we investigated whether anti-miR-214 could rescue the SMC differentiated phenotype in previously de-differentiated cells from PAH patients. Indeed, antagonism of miR-214 in PAH-PASMC reversed the depressed expression of MEF2C (Fig 7A) and LMOD1 (Fig 7B), suggesting reversion to the contractile phenotype. Moreover, we examined the effect of miR-214 antagonism on PAH-PASMC proliferation. Depicted in Fig 7C, antagonism of miR-214 in PAH-PASMC is likely to explain a brake in the cell cycle progression and attenuated cell proliferation.

**Fig 8:**
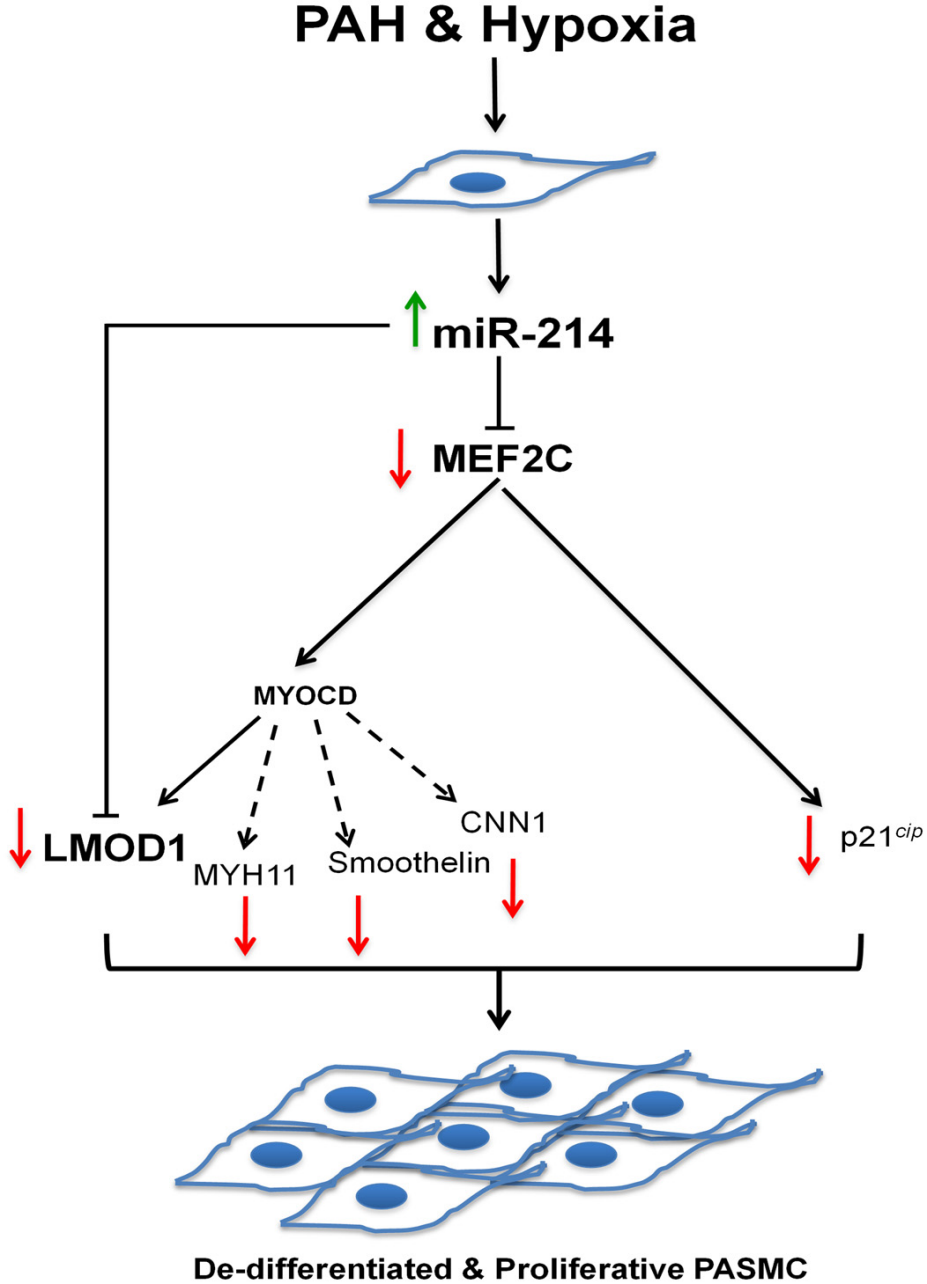
Schematic of miR-214-mediated effects on PAH-related and hypoxia-induced SMC phenotypic modulation and de-differentiation. During vascular homeostasis, SMCs maintain a contractile/differentiated phenotype, which changes to a pro-proliferative, synthetic or de-differentiated phenotype upon injury. Hypoxia-induced upregulation of miR-214 exerts direct suppression both at the level of MEF2C and LMOD1 to mediate phenotype switching and pro-proliferative response associated with vascular remodeling.

Fig 8 illustrates the proposed mechanism by which PAH and hypoxia induce miR-214 and direct (via MEF2C) phenotype change from a contractile to non-contractile, “synthetic” pro-proliferative state.

## Discussion

To our knowledge, these findings are the first to describe a complex orchestration of SMC signaling and de-differentiation by miR-214 in human PAH explants and cells exposed *in vitro* to hypoxia. Here, we show that miR-214 is upregulated in human PAH and controls key upstream modulators of the differentiated contractile SMC phenotype. Hallmark identifiers of the differentiated contractile phenotype, MEF2C, MYOCD and LMOD1 were downregulated in human PAH and/or SMCs exposed *in vitro* to hypoxia. Based on a strong association of miR-214 with MEF2C and contractile protein LMOD1 in PAH, and exact complementarity of the miR in their 3’UTR regions, we tested miR-214’s potential synergistic role in suppressing MEF2C (and attendant downstream contractile proteins, Fig 8) and LMOD1. Indeed, overexpression of miR-214 downregulated expression of MEF2C, LMOD1 and other MYOCD-regulated contractile proteins, such as MYH11, smoothelin and CNN1. Reciprocally, miR-214 antagonism restored the depressed expression of MEF2C and LMOD1 (as well as other contractile proteins) in SMCs subjected to hypoxia. Mutagenesis and reporter assays confirmed that both LMOD1 and MEF2C are direct and functional targets of miR-214, binding directly to their 3’UTR. These results together demonstrate that miR-214 directly modulates expression of contractile protein at the level of LMOD1 as well as *via* upstream disruption of MEF2C-MYOCD signaling. Intriguingly, antagonism of miR-214 also reversed hypoxia-induced suppression of cell cycle inhibitor p21^*cip*^ and abrogated cell proliferation. On the other hand, miR-214 mimic promoted these processes under normoxic conditions. Finally, decreases in MEF2C and LMOD1, and increases in proliferation of PAH patient cells were reversed by the antagomir. Taken together, these findings appear to advance a synergistic inhibition by miR-214 at parallel and linear checkpoints of differentiation and propose miR-214 antagonists as a potential new therapy to mitigate PAH-related and other vasculoproliferative disorders.

The structure, function and tone of the pulmonary vascular bed depend characteristically on the contractility of the smooth muscle cells. However, in response to a vascular injury, SMCs undergo phenotypic switching to a de-differentiated phenotype and become proliferative [1,6]. It is broadly accepted that MYOCD, expressed specifically in cardiac and SMCs, is the most important transcription factor necessary for activation of smooth muscle differentiation. Several studies have now shown that MYOCD associates with the MADS (MCM1, Agamous, Deficiens, Serum Response Factor)-box of SRF and activate expression of several critical gene products involved in proliferation, migration and contractile function of SMCs [13,34]. Interestingly, MEF2C, another cardiac and smooth muscle-specific transcription factor shown to be pivotal for SMC differentiation, positively regulates MYOCD [13,14,35]. Our findings show that MEF2C and MYOCD are markedly suppressed in PAH-PASMCs. In this study, we also provide evidence to support miR-214-mediated downregulation of MEF2C activity in smooth muscle cells. These results are consistent with recent studies showing that knockdown of MEF2C causes proliferation in pulmonary endothelial cells [36]. That said, future studies could explore whether miR-214 is an important post-transcriptional regulatory control for endothelial cells in PAH. At this juncture, it is critical to point out that, on the one hand, these data run contrary to some of findings in the literature that imply that hypoxia can induce MYOCD via a hypoxia response element in the MYOCD promoter [37,38]. On the other hand, our findings are congruent with others that display precisely the opposite effect [4]. Whereas the latter scenario of an inverse relationship between hypoxia and MYOCD would explain SMC de-differentiation and hyperplasia in our study, the differential response across studies is likely to be species-, tissue- and context-dependent as well as in areas of the lung more likely to harbor progenitor cells in a hypoxic environment [39].

Leiomodin1 (LMOD1) is a novel SMC-specific gene, which is under the transcriptional regulation of MYOCD [16]. Although the precise role of LMOD1 in the physiological setting is yet to be completely characterized, it has been implicated in SMC contractile activity and actin cytoskeleton assembly via its association with tropomyosin [16]. Our results are the first to our knowledge to show that LMOD1 expression is significantly attenuated both in the pulmonary arteries and lungs, and is clinically relevant in the hPASMCs of PAH patients.

While a differentiated, contractile SMC phenotype is a critical factor in vascular homeostasis, the molecular mechanisms involved in its modulation remain elusive. MEF2-dependent transcription is known to be regulated by activators such as p38 MAP kinase[40], Ca^2+^/calmodulin kinases (CaMKs)[41], and repressors including, sumoylation[42], and histone deacetylases[43]. The current study is unique in showing miRNA-dependent attenuation of MEF2 in vascular SMCs, and hence SMC phenotype modulation. Here, we demonstrate that miR-214 is an upstream regulator of MEF2C (and by extension MYOCD) as well as LMOD1. Further, *in silico* analyses using miRBase showed that miR-214 has two potential binding sites in the LMOD1 3’-UTR, and one in the MEF2C 3’-UTR, which are conserved among humans, chimpanzees, rhesus monkeys, rats and mice, and are broadly conserved among vertebrates. Human miR-214 is transcribed as a member of the miR-199a-214 cluster, which, interestingly, is regulated in a HIF1α- and/or HIF-independent manner [27]. A recent study reported miR-214-mediated cardiac hypertrophy [44], supporting observations that miR-214 is upregulated in heart failure patients [28,45], a negative consequence associated with PAH. Furthermore, mice with experimental deletion of the miR-199/214 locus display defects in skeletal muscle formation [24,46], suggesting a pivotal role of miR-214 in myogenesis. Our study, however, provides a new perspective on the role for miR-214 in human vascular smooth muscle cell biology. In accordance with this, we found that miR-214 is significantly upregulated in hPASMC-derived from PAH cohort and control cells exposed to hypoxia (Fig 2B and 2C), suggesting a critical role for miR-214 in the pathophysiological changes associated with PAH.

Although miR-214 has been shown to be involved in the regulation of multiple target genes, such as PPARδ [27], Ncx1[26], Ezh2[25,44], ATF4[47], our findings indicate that miR-214 participates in the heretofore undescribed regulation of a MEF2C-MYOCD-contractile protein expression pathway, which we prove to be essential for vascular homeostasis and phenotypic modulation of SMC. As a key factor in driving SMC phenotypic changes associated with PAH, hypoxia is known to stimulate SMC proliferation, migration, and resistance to apoptosis leading to increased medial thickening and extensive remodeling of the vessel wall [1,4]. Herein, we show that hypoxia-mediated upregulation of miR-214 accounts for the downregulation of SMC contractile proteins, MEF2C, MYOCD, LMOD1, MYH11, smoothelin and CNN1 in control hPASMC (Fig 5A–5F), resulting in medial thickening of the vessel wall, as is evident from S2A Fig.

In addition, we demonstrated that hypoxia-induced SMC proliferation is attenuated by miR-214 antagomiR. The hypoxia-induced increase in proliferation coincides with progression of SMCs to S and G2/M phases and a concomitant decrease in G1/G0 to S-phase cell cycle progression inhibitor, p21^*cip*^ *(*Fig 6). This suggests that miR-214 could promote SMC proliferation by regulating cell cycle progression via targeting at a third level, cyclin-dependent kinase inhibitor, p21^*cip*^ (Fig 8). Indeed, this assertion is supported by a recent study associating the downregulation of MEF2C expression to repression of p21^*cip*^ levels, thus leading to an augmentation of cells in S phase and overall cell proliferation [48]. With that information in mind, our findings suggest that PAH and hypoxia-induced upregulation miR-214 and subsequent suppression MEF2C would disinhibit p21^*cip*^. This, in turn, could be expected to release a putative “brake” on cell cycle progression and permit SMC proliferation. Whether or not p21^*cip*^ could be more directly inhibited by miR-214 requires further study.

Furthermore, we demonstrate that ectopic increases in miR-214 in control hPASMC mimics a PAH phenotype, characterized by suppression of the MEF2C-MYOCD-LMOD1 axis, as well as other SMC-specific contractile proteins, including MYH11, smoothelin and CNN1 and, increased hPASMC proliferation and cell cycle progression (Figs 4 and 6). Overall, we provide strong evidence that miR-214 is important for SMC phenotype regulation in the shift from a contractile to hyperplastic response to hypoxia and PAH.

Finally, a major highlight of the present study is the potential for miR-214 as a therapeutic target in vascular disease. We demonstrate for the first time that inhibition of miR-214 by exogenous administration of anti-miR-214 (miR-214 antagomiR) in hPASMC-derived from PAH patients rescues the contractile phenotype of these cells. This is supported by increased expression of both MEF2C and LMOD1 upon treatment of hPASMC with anti-miR-214. It has been shown in recent studies that miR-214 is upregulated in other cardiovascular disorders [47], further underscoring the potential of miR-214 as a therapeutic target. That modulation of miR-214 can singularly intervene at multiple sites in the contractile phenotype cascade (i.e. MEF2C and LMOD1) and silence broad contractile protein expression provide a firm rationale for it as a potential therapeutic target.

In summary, we provide compelling evidence that miR-214 plays a pivotal and pleiotropic role in modulating SMC differentiation and proliferation by multi-tiered targeting of the MEF2C-MYOCD-LMOD1 signaling pathway, and at the level of p21^*cip*^. Indeed, we demonstrate PAH-induced miR-214 effects SMC de-differentiation by direct translational targeting of LMOD1, and provide strong evidence of the same for MEF2C. Our observations indicate that miR-214-MEF2C-LMOD1 interactions form a novel nexus of regulation of SMC phenotype associated with PAH. They are therefore, likely to inform novel therapeutic interventions for treatment of cardiovascular diseases including pulmonary arterial hypertension.

## Supporting Information

S1 Fig Quantitative RT-PCR analysis was performed on PA homogenates to quantify mRNA expression. mRNA expression of MEF2C (A), MYOCD (B), LMOD1 (C), MYH11(D), smoothelin (E) and CNN1 (F) is attenuated in PA from PAH patients compared to non-PAH control subjects (*n* = 3).
(TIF)

S2 Fig (**A, B**). Smooth muscle contractile proteins, LMOD1, smooth muscle α-actin and smoothelin are downregulated within PAs of PAH lungs. LMOD1, smooth muscle α -actin and smoothelin expression in lungs from PAH and non-PAH subjects (*n = 3*) were determined using immunofluorescence (IF). Co-localization and marked decrease in LMOD1 (red) and smooth muscle α-actin (green) expression in the vessel wall of PAH samples compared to the non-PAH group (A). Images from lung sections also showed colocalization and reduced expression of LMOD1 (red) and smoothelin (green) in the vessel wall of PAH samples compared to the non-PAH group (B).
(TIF)

S3 Fig miR-214 binding sites in LMOD1 and MEF2C 3’UTR are conserved among vertebrates. (TIF)

S4 Fig miR-214 mediates hypoxia-induced proliferation of hPASMCs. hPASMC transfected with anti-miR-214 or scrambled control were exposed to normoxia or hypoxia for 24 hrs and then lysed for BrdU incorporation assay by ELISA. Treatment with anti-miR-214 significantly attenuated hypoxia-induced proliferation. (*n = 9–12*). Graphs represent mean ± SEM (*p<0.05; ** p<0.01).
(TIF)

S5 Fig Increased proliferative potential of PAH-PASMCs. Cell Cycle Analysis was performed using flow cytometry assay on hPASMCs-derived from non-PAH and PAH cohorts. The number of proliferating PASMCs was significantly higher (~1.8 fold, p<0.05) in PAH samples compared to non-PAH subjects. (*n = 3*). Graphs represent mean ± SEM (*p<0.05; ** p<0.01).
(TIF)

S1 File Supplemental Information on Methods. (DOCX)

S2 File List of Abbreviations. (DOCX)
