# Supplementary figures and images for: MEF2C-MYOCD and Leiomodin1 Suppression by miRNA-214 Promotes Smooth Muscle Cell Phenotype Switching in Pulmonary Arterial Hypertension
Source: PLoS One. 2016 May 4;11(5):e0153780. doi: 10.1371/journal.pone.0153780 (PMC4856285; doi:10.1371/journal.pone.0153780)

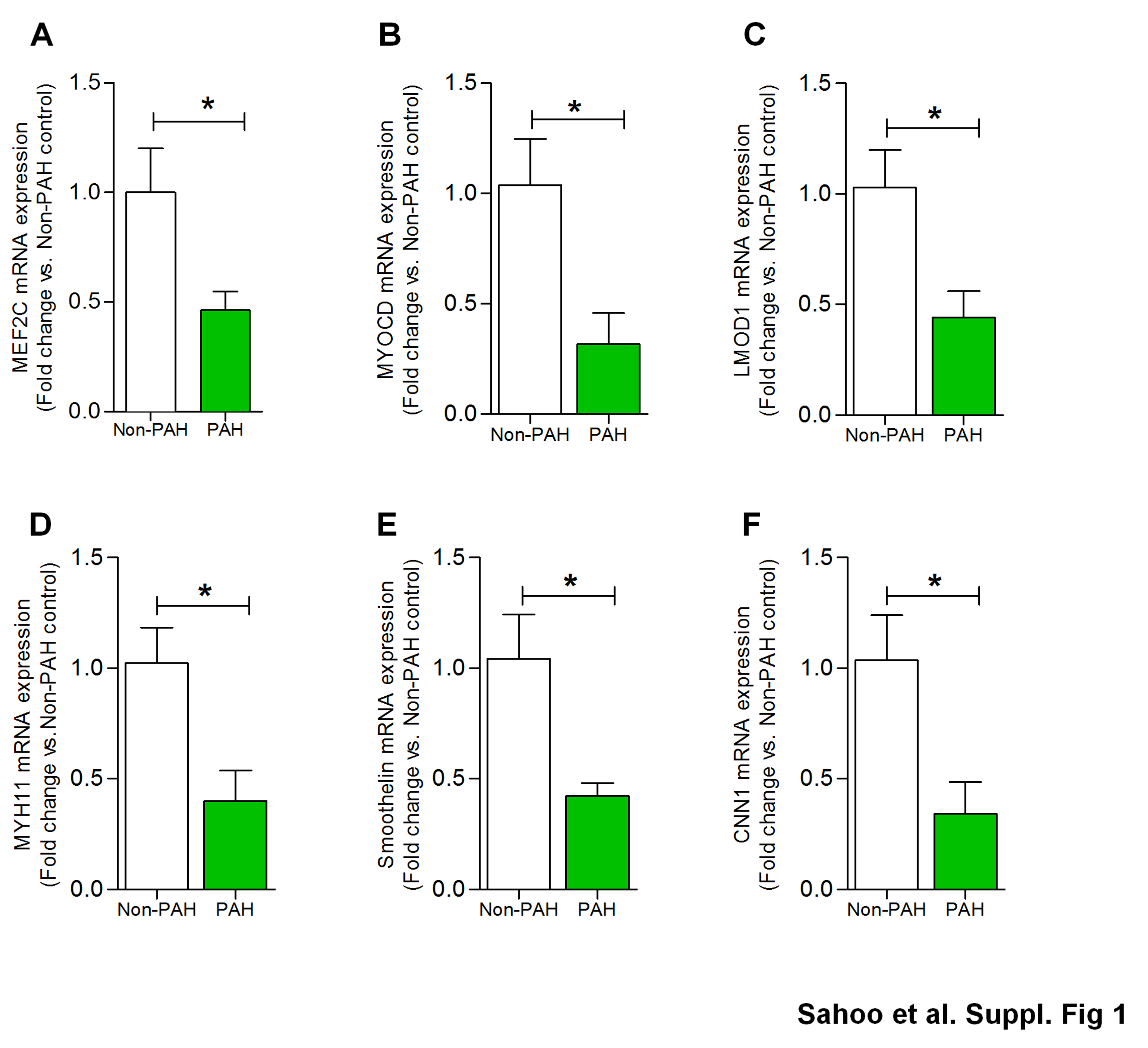

Supplement: S1 Fig — mRNA expression of MEF2C (A), MYOCD (B), LMOD1 (C), MYH11(D), smoothelin (E) and CNN1 (F) is attenuated in PA from PAH patients compared to non-PAH control subjects (n = 3). (TIF) [file pone.0153780.s001.tif]

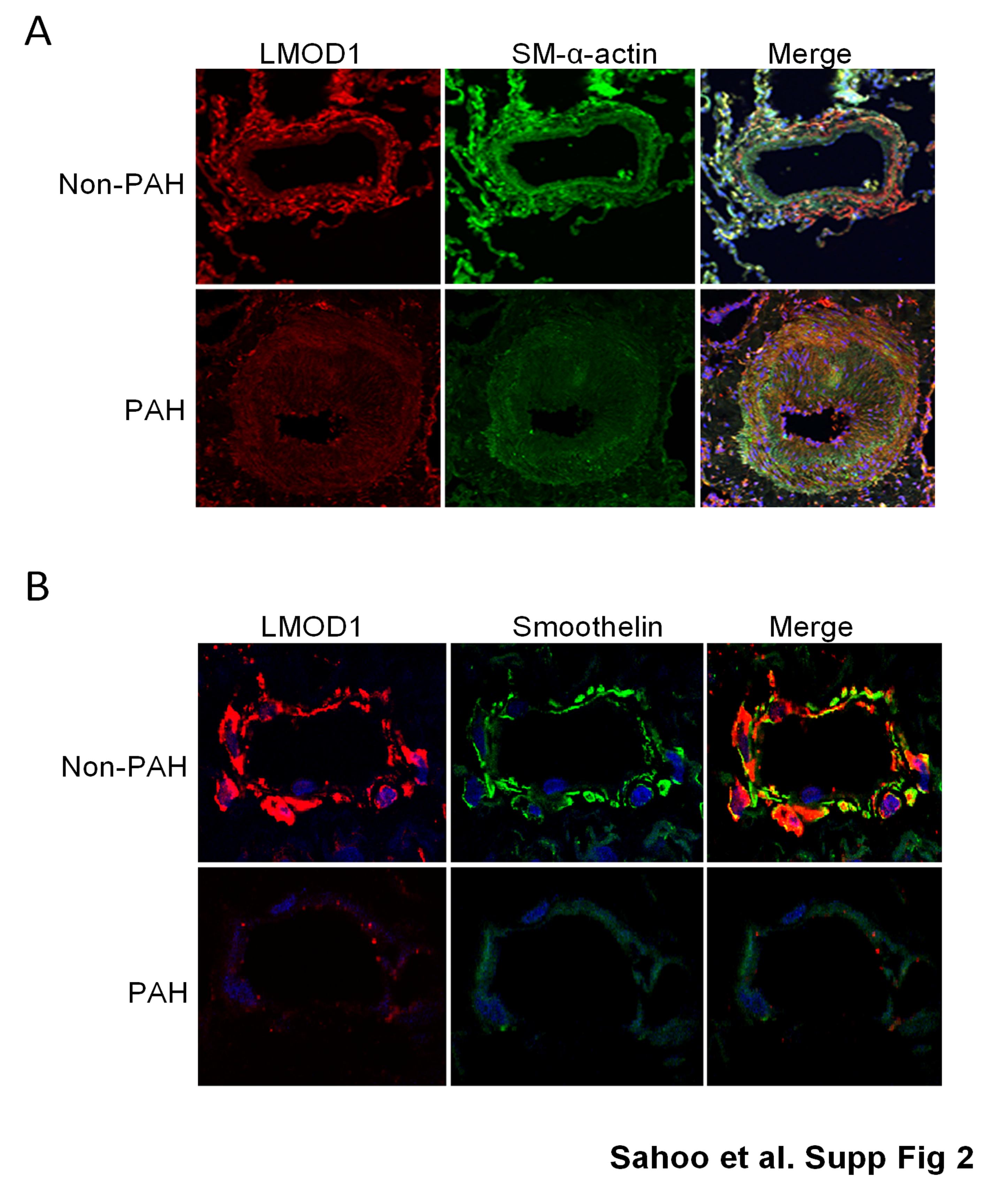

Supplement: S2 Fig — (A, B). Smooth muscle contractile proteins, LMOD1, smooth muscle α-actin and smoothelin are downregulated within PAs of PAH lungs. LMOD1, smooth muscle α -actin and smoothelin expression in lungs from PAH and non-PAH subjects (n = 3) were determined using immunofluorescence (IF). Co-localization and marked decrease in LMOD1 (red) and smooth muscle α-actin (green) expression in the vessel wall of PAH samples compared to the non-PAH group (A). Images from lung sections also showed colocalization and reduced expression of LMOD1 (red) and smoothelin (green) in the vessel wall of PAH samples compared to the non-PAH group (B). (TIF) [file pone.0153780.s002.tif]

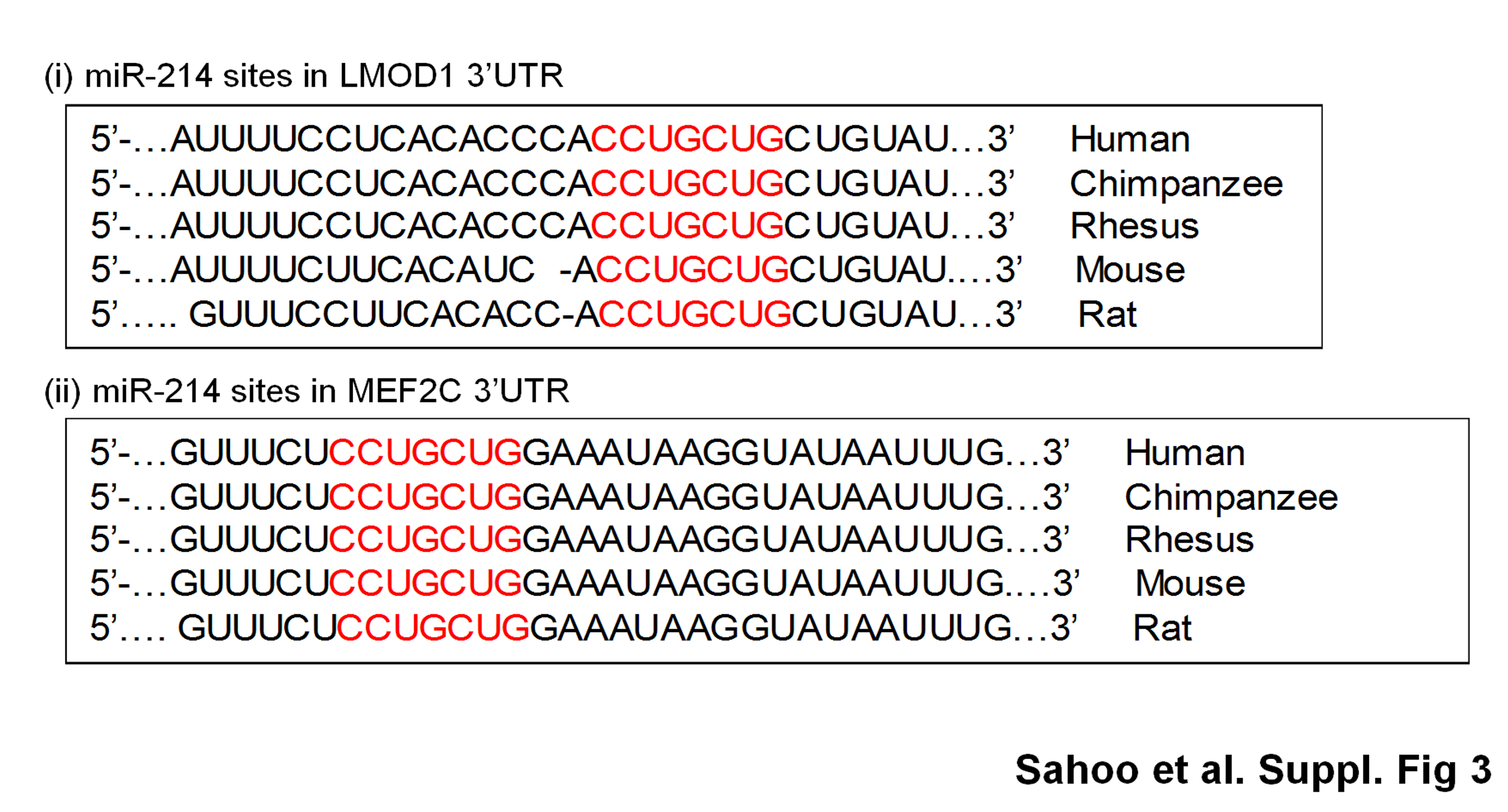

Supplement: S3 Fig — (TIF) [file pone.0153780.s003.tif]

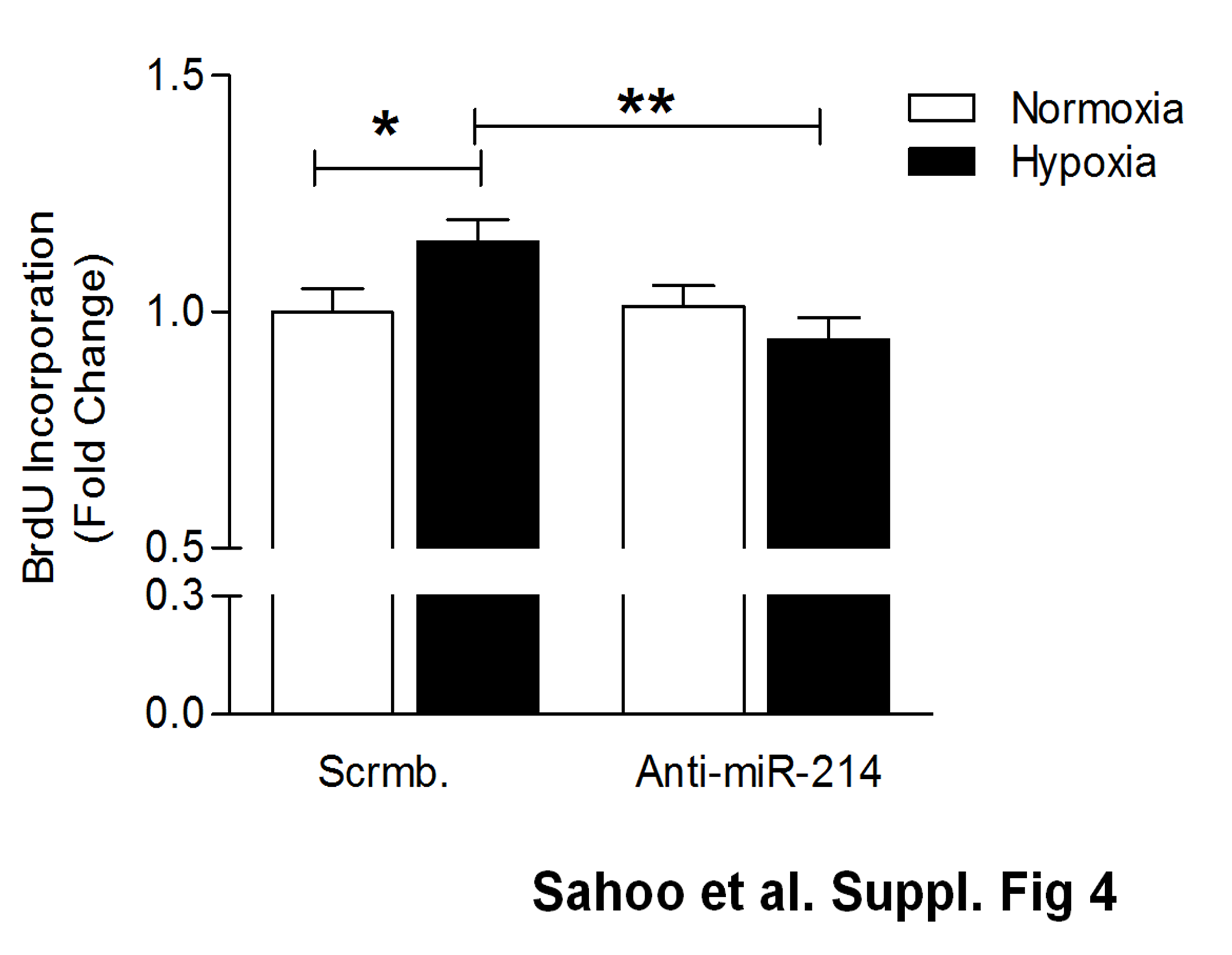

Supplement: S4 Fig — hPASMC transfected with anti-miR-214 or scrambled control were exposed to normoxia or hypoxia for 24 hrs and then lysed for BrdU incorporation assay by ELISA. Treatment with anti-miR-214 significantly attenuated hypoxia-induced proliferation. (n = 9–12). Graphs represent mean ± SEM (*p<0.05; ** p<0.01). (TIF) [file pone.0153780.s004.tif]

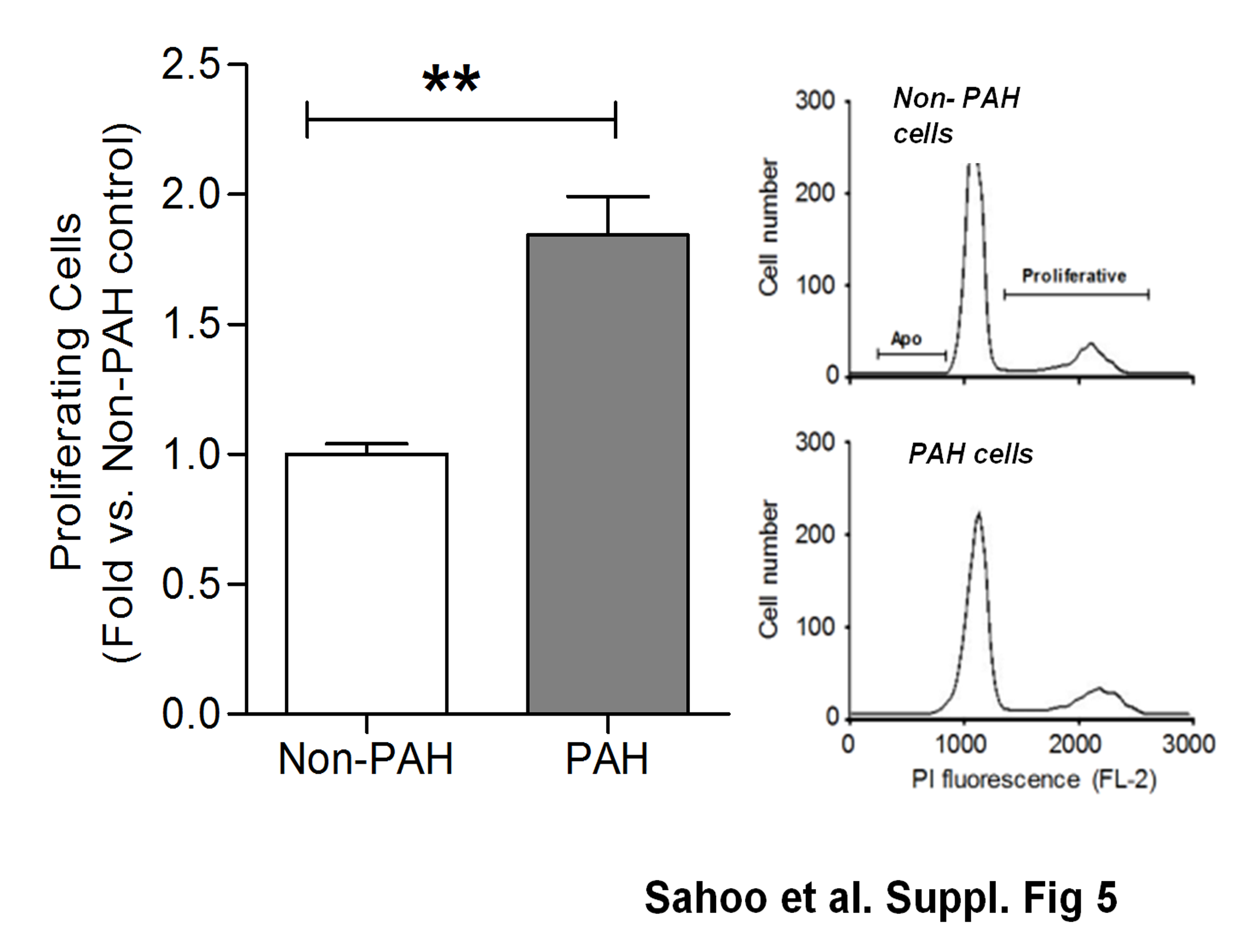

Supplement: S5 Fig — Cell Cycle Analysis was performed using flow cytometry assay on hPASMCs-derived from non-PAH and PAH cohorts. The number of proliferating PASMCs was significantly higher (~1.8 fold, p<0.05) in PAH samples compared to non-PAH subjects. (n = 3). Graphs represent mean ± SEM (*p<0.05; ** p<0.01). (TIF) [file pone.0153780.s005.tif]
