# Supplementary material for: MEF2C-MYOCD and Leiomodin1 Suppression by miRNA-214 Promotes Smooth Muscle Cell Phenotype Switching in Pulmonary Arterial Hypertension
Source: PLoS One. 2016 May 4;11(5):e0153780. doi: 10.1371/journal.pone.0153780 (PMC4856285; doi:10.1371/journal.pone.0153780)
Supplement: S1 File — (DOCX) [file pone.0153780.s006.docx]

***SUPPLEMENTAL MATERIAL***

***Methods***

***Western Blot***

Proteins isolated from cultured cells or tissue homogenates were analyzed by Western blot analysis as described previously^1^. Antibodies to leiomodin1 (Protein Tech), calponin 1, smoothelin, β-actin (Santa Cruz), myocardin (R& D Systems, Santa Cruz), MYH11 (Abcam), MEF2C, p21*^cip^*, (Cell Signaling) were added to the blots for overnight incubation followed by anti-mouse or anti-rabbit secondary antibodies conjugated to horseradish peroxidase (Bio-Rad). The enhanced chemiluminescence (ECL) Western blotting detection system (Pierce) was used to facilitate detection of protein bands. Protein densitometry was determined using Image J software (NIH)^2^.

***Immunofluorescence***

For immunofluorescent labelling of lung and pulmonary artery (PA) sections, 5 µm sections of paraffin-embedded lungs and PA were antigen-retrieved, fixed in 2% paraformaldehyde, permeabilized with 0.1% Triton X-100 and washed using PBS. Sections were blocked in 2% BSA/PBS solution for 30 min at RT. Slides were incubated with anti-LMOD1 or anti-SM actin antibody (1:200) followed by FITC- or Cy3-conjugated secondary antibody (1:500), respectively (Life Technologies *Inc*.). Slides were stained for nuclei (DAPI), and cover-slipped using gelvatol mounting media (polyvinylalcohol, glycerol, H_2_O, sodium azide and Tris pH 8.5). Nonspecific rabbit or goat IgG (5 μg/ml) was used in lieu of primary antibody as a negative control. Confocal images were captured on an Olympus Fluoview 1000 confocal microscope (Olympus America Inc., Bethlehem, PA). For each experiment 5–8 images per treatment group were captured. Three independent experiments were performed.

***Cell Proliferation Assay and Cell Cycle Analysis***

Proliferation of hPASMC cells was measured by counting viable cells upon trypan blue staining as described^3^, and by BrdU incorporation using ELISA. For BrdU incorporation, hPASMC transfected with antagomir or control were labeled with 10 μM BrdU (Sigma) and exposed to 21% or 1% O_2_ for 24 hrs. Equal amounts of protein were loaded on 96-well plates in binding buffer and incubated overnight at 4^◦^C. BrdU incorporation was monitored by direct ELISA per the manufacturer’s instructions (Pierce Bio.). The difference in absorbance 405 and 650 nm was expressed as fold change vs. normoxic controls. Cell cycle analysis was performed as follows: SMC were harvested and diluted in PBS followed by drop-wise addition of 100% ice-cold ethanol to make a final 70% ethanol/PBS fixative solution and stored overnight at −20°C. Cells were washed twice with cold PBS, pelleted at 300 x *g*, and resuspended in propidium iodide (50 µg/ml)/ribonuclease A (250 µg/ml) in PBS solution for 45 minutes at 37°C. Following PBS washing, samples were run on a flow cytometry system (FACS Calibur; BD, San Jose, USA) and cell cycle profiles analyzed using FlowJo software.

**References:**

1. Ranayhossaini DJ, Rodriguez AI, Sahoo S, Chen BB, Mallampalli RK, Kelley EE, Csanyi G, Gladwin MT, Romero G and Pagano PJ. Selective recapitulation of conserved and nonconserved regions of putative NOXA1 protein activation domain confers isoform-specific inhibition of Nox1 oxidase and attenuation of endothelial cell migration. *J Biol Chem*. 2013;288:36437-50.

2. Schneider CA, Rasband WS and Eliceiri KW. NIH Image to ImageJ: 25 years of image analysis. *Nat Methods*. 2012;9:671-5.

3. Doyle A, Griffiths, J.B., and Newell, D.G. *Cell and Tissue Culture: Laboratory Procedures.*: John Wiley & Sons, Inc., Chichester, England.; 1995.
