# Supplementary material for: MEF2C-MYOCD and Leiomodin1 Suppression by miRNA-214 Promotes Smooth Muscle Cell Phenotype Switching in Pulmonary Arterial Hypertension
Source: PLoS One. 2016 May 4;11(5):e0153780. doi: 10.1371/journal.pone.0153780 (PMC4856285; doi:10.1371/journal.pone.0153780)
Supplement: S2 File — (DOCX) [file pone.0153780.s007.docx]

**List of Abbreviations:**

BrdU 5-Bromo-2’-deoxyUridine

CNN1 Calponin 1

hPASMC human pulmonary artery smooth muscle cell

LMOD1 Leiomodin1

miRs MicroRNAs

MEF2C Myocyte Enhancer Factor 2C

MYOCD Myocardin

MYH11 Smooth Muscle Myosin Heavy Chain

PA Pulmonary Artery

PAH Pulmonary Artery Hypertension

SMC Smooth Muscle Cell

UTR Untranslated region
